# Fortification of *Carica papaya* fruit seeds to school meal snacks may aid Africa mass deworming programs: a preliminary survey

**DOI:** 10.1186/s12906-018-2379-2

**Published:** 2018-12-07

**Authors:** M. Kugo, L. Keter, A. Maiyo, J. Kinyua, P. Ndemwa, G. Maina, P. Otieno, E. M. Songok

**Affiliations:** The Graduate School, Kenya Medical Research Institute, Mbagathi Road, Nairobi, P.O. BOX 54840 Kenya

**Keywords:** *Carica papaya*, Albendazole, Mass drug administration, Helminths, Ringworms, School children, Porridge, Africa, Child nutrition

## Abstract

**Background:**

Soil transmitted helminths (STHs) are among the world’s neglected tropical diseases. Morbidity due to STHs is greatest in school-age children who typically have the highest burden of infection. In 2001, WHO passed a resolution for the use of large-scale mass drug administration (MDA) to deworm vulnerable children through school based programs. Though effective, there is concern that MDA might not be sustainable over extended periods. Additionally the current MDA strategy does not consider child malnutrition, a very common malady in resource limited countries. We report a pilot evaluation of an innovation that bundles school feeding and deworming.

**Methods:**

We designed a maize (corn) flour fortified with grounded dried papaya (*Carica papaya*) seeds and used it to prepare porridge as per the usual school meal recipe Children from three primary schools from Nandi County in Kenya were randomized into three arms: One school received 300 ml papaya fortified porridge daily (papaya group), the second school received similar serving of plain porridge without the pawpaw ingredient (control group) and the third school received plain porridge and the conventional MDA approach of one time 400 mg dosage of albendazole (albendazole arm). Prior to the randomization, an initial baseline stool microscopy analysis was done to determine presence and intensity of intestinal worms. Core indicators of nutrition-height, weight and hemoglobin counts were also assessed. The children were monitored daily for two months and final stool sample analysis and clinical monitoring done at the end of the study. Baseline and follow-up data were analyzed and compared through SAS version 9.1 statistical package.

**Results:**

A total of 326 children participated in the trial. The overall prevalence of *Ascaris lumbricoides* was 29.4% (96), *Trichuris Trichura* 5.2% (17) and hookworm 1 (0.3%). Papaya seed fortified porridge reduced the *Ascaris lumbricoides* egg count by 63.9% after the two month period (mean 209.7epg to 75.7 *p* < 0.002) as compared to the albendazole arm 78.8% (129.5 epg to 27.5, *p* value 0.006). The control group showed an increase in egg count (42.epg to 56.3) though it was not statistically significant. Hemoglobin counts in the papaya group increased from a mean of 2 g/dL (11.5 g/dL to 13.5 g/dL, *p* < 0.001), as compared to the albendazole arm that increased by 1 g/dL (12.8–13.9, *p* < 0.001). No significant change was observed in the placebo arm (13.2 to 13.1). Interestingly the papaya group showed a significant reduction of children with *Tinea capitis* (ringworms) (54.4 to 34%, *p* < 0.002) as compared to the albendazole arm that showed an increase in ringworm infestation though not statistically significant (39.7 to 64.7% *p* = 0.608).

**Conclusion:**

Papaya seed fortified porridge had a significant effect on reduction of *Ascaris lumbricoides* burden. It had a better nutritional outcome and effect on child fungal infections than albendazole. Its application as a routine school meal may aid current national school based nutrition and deworming programs in Africa.

**Trial registration:**

This study was retrospectively registered at Clinicaltrials.gov Ref. NCT02725255 on 31st March 2016.

## Introduction

Infections by intestinal parasites including the soil transmitted helminths (STH: *Ascaris lumbricoides, Ancylostoma duodenale (*hookworm) *and Trichuris Trichura*) and schistosomiasis are an important public health concern [1]. These infections are most prevalent in poor settings of the developing world with the highest burden of infection registered among school-aged children [2]. They are commonly associated with malnutrition, anaemia, impaired growth, poor school attendance and impaired cognition [3–6]. In 2001, the World Health Assembly passed a resolution for endemic countries to implement large-scale mass drug administration (MDA) of school-aged children using albendazole chemotherapy to reduce the burden of STH infections [1].

While school based MDA programs have significantly contributed to reducing the burden of infection by intestinal worms among school children, several concerns still exist over the large-scale use of chemotherapeutic anthelminthic drugs in deworming. The large population of children and the high frequency of dosage may pose a challenge on the sustainability of these programs. Further, the MDAs exert increasing drug pressure on parasite populations, a circumstance that is likely to favour parasite genotypes that can resist chemotherapy [7, 8]. Additionally, the current MDA strategy does not consider child malnutrition, a widespread problem in most resource limited countries. There is hence a need to design treatment alternatives that are not only affordable and sustainable but easier to implement with a minimal chance of development of resistance.

Components of the tropical fruit, *Carica papaya* (pawpaw), have been scientifically proven to have potent anthelminthic and antiamoebic properties [9, 10]. Its seeds have been demonstrated in various studies to have anthelminthic properties [11, 12]. Follow- up phytochemical analysis show that benzyl isothiocyanate is the bio-active anthelminthic ingredient in the seeds [13]. A study conducted in Nigeria showed that air dried papaya seeds given as elixir with honey significantly reduced intestinal parasites among school children without any side effects [14]*.* As a food source, with no known harmful effects, the above evidence suggests that papaya seed extracts can be a sustainable candidate for deworming school children.

Based on the above, we have designed and tested an alternative MDA approach which integrates deworming into the school feeding program. We have included ground papaya seeds to daily school meals through compounding it with maize flour, a common ingredient for school nutrition in sub-Saharan Africa.

## Materials and methods

### Product development

Maize, millet and pawpaw seeds were mixed in a ratio of 5:1:2.5 and ground together into flour using a hammer mill. Micronutrient fortificant (DSM, South Africa) was then added at an inclusion rate of 0.25%. The micronutrient premix supplied nutrients as reported in Table 1. Porridge was prepared by heating water up to boiling and adding the product, stirring for some minutes then adding sugar. Each child received 300 mL of the porridge every day, constituting a dose of 10 g of the *Carica papaya* seeds per child per day.

**Table 1:** Nutrient Composition of Micronutrient Fortificant Used in the Study

Nutrient	Amount per Kg of fortificant
Vitamin A (IU)	4,162,500
Iron (mg)	40,000
Zinc (mg)	60,000
Thiamine (mg)	8000
Riboflavin (mg)	7000
Niacin (mg)	61,000
Vitamin B6 (mg)`	10,000
Folate (mg)	3000
Vitamin B12 (μg)	14,000

### Study design

The study was a placebo-controlled trial carried out from May to August 2015 and involved primary school children in Nandi County, Kenya. Based on earlier STH prevalence surveys, ten schools in the area were surveyed and three were selected to participate in the study. The schools-Mosine, Soiyet and Barasendu-had a total pupil population of 1074. Through sample size calculation a total 324 children were recruited to the study. At least one hundred school pupils were then randomly selected using random number generator (random.org) from each of the participating schools to represent all pupil age range in the school (Randomisation was carried out by MK). The schools were assigned randomly to one of three treatment groups: children either received 400 mg albendazole (Zentel ®, GlaxoSmithKline) to form the albendazole group, maize meal porridge with pawpaw seeds (papaya group) or maize meal porridge without pawpaw seeds (control group). Children in the albendazole group also received plain maize meal porridge without pawpaw seed. Before enrolment, parents/guardians of children in the selected schools were given a comprehensive explanation of the trial including the risks and benefits involved and written informed consent was obtained. To reinforce community confidence on the product and the clinical trial process, the fortified flour was prepared and mixed at their local milling plant. The study was approved by the Kenya Medical Research Institute, Ethical Review Committee on 18th June13, 2013 (Ref KEMRI SSC 2580) and an annual renewal sought and approved from 16th September 2014 until July 2016. Participant recruitment for this study began in March 19th 2015. Baseline and follow-up data collection was conducted during the second term of the school year from May 11th to August 6th 2015.

The study is registered under ClinicalTrials.gov (ID: NCT02725255). Registration of the trial was done retrospectively for investigators were not aware that such preliminary proof of principle data fell under a category that required registration. The authors confirm that all ongoing and related trials for this intervention are registered.

**Fig. 1:**
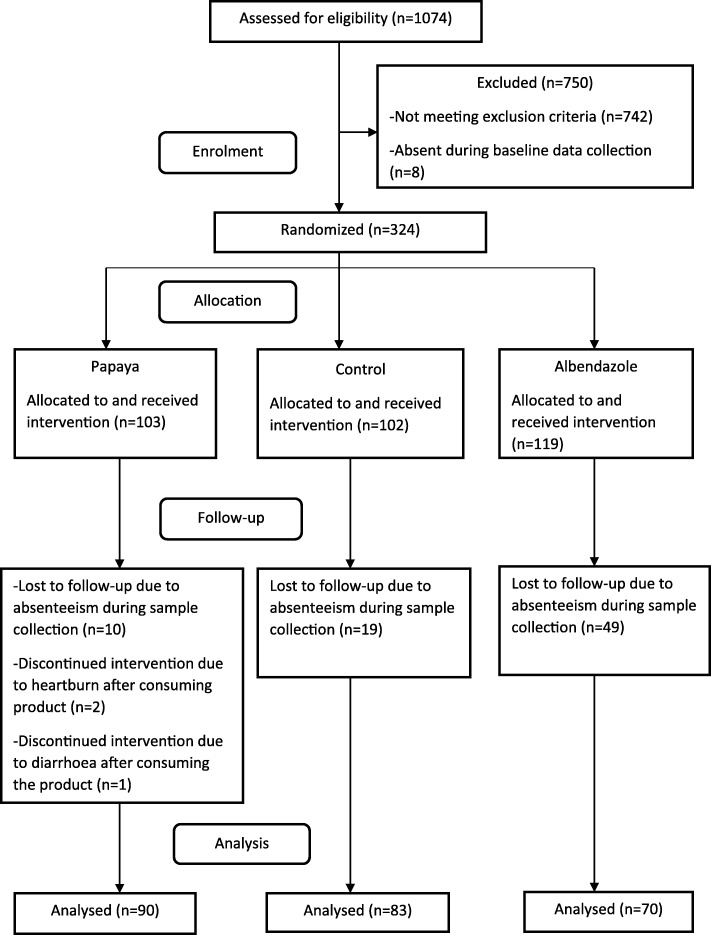
The flow chart summary of the study design

The flowchart summary of the study design is shown in Fig. 1.

### Eligibility criteria and baseline assessment

The inclusion criteria included; male or female, age 4–12 years and informed consent. The exclusion criteria were previous hypersensitivity to albendazole or pawpaw products, receipt of any anthelminthic in the two weeks before enrolment and during the study. During the baseline, structured questionnaires were administered to collect health and socio-demographic data. Anthropometric measurements were taken and stool and blood samples collected for each participating child. Participants were assessed for presence of ringworms and other skin infections.

### Treatment

After baseline data collection, albendazole was offered as a single dose of 400-mg chewable tablet to all children in the control group. All the children in the placebo and control groups received plain porridge everyday throughout the study period. All eligible children in the test group were given 300 mL of the porridge fortified with the pawpaw seeds daily at break time (10 am) for two months. Due to funding limitations, trial follow-up was stopped after two months. At the end of the two month period a repeat stool, blood and anthropometric measurements were taken from each participating child. After completion of the study, all children in the test group and placebo arm were treated with 400 mg albendazole.

### Parasitology

For stool sample collection, children were given 125 ml transparent plastic containers and asked to collect a small portion of their faecal samples which were transported to the laboratory for parasite ova and cysts examination including faecal egg count analysis. For pre- and post- treatment examination, faecal egg counts were assessed within six hours of being produced. Stool samples were analysed using the Kato-Katz and formal-ether concentration (FEC) methods according to the WHO guidelines [15]. The smears were prepared in duplicate and read by two different technicians to ensure the accuracy and precision of egg counts. A random sample of 10% of the smears prepared was taken to a different laboratory for quality control. Slides were re-examined for the quality control difference in egg counts. The cure rate and egg reduction rate was determined through the comparison of egg counts pre- and post-treatment.

### Haemoglobin

Capillary blood samples were collected by finger prick from each child by piercing the fingertip using an automated lancet. Haemoglobin concentration was measured directly by use of the Hemocue.

### Anthropometry

Anthropometric measurements were taken by the same field worker at baseline and at the two follow up data collection points. The heights of the children were measured without shoes to the nearest 0.1 cm using a wooden height board. Children’s weights were measured to the nearest 100 g using a portable battery powered digital scale with children wearing standard school clothing without footwear according to standard procedures. Mid upper arm circumference (MUAC) was measured to the nearest 0.1 cm and triceps skin fold (TSF) thickness were measured using standard methods. The height, weight and age of the children were used to calculate the body mass index (BMI) for age.

### Statistical analysis

Data were analysed using SPSS version 13.0 software (SPSS, Inc., Chicago, IL). The parasite infection prevalence, defined as the percentage of children with eggs in faeces was calculated at baseline and at follow up. Parasite eggs counted are expressed as eggs/g of faeces. Initial parasite load was corrected for by including it in the analysis of variance. Only individuals for whom there were complete data on infection status from baseline to week 8 were included in this analysis.

## Results

### Baseline characteristics

Of the 324 eligible children recruited at baseline, complete sets of data was obtained for an overall of 243 subjects; 90 in the papaya group, 83 in the albendazole group and 70 in the control group (Table 2). Reasons for drop-out included transfer to different schools, absenteeism and inability to produce stool samples. During the sampling day, the mean ± SD age of participants was 8.7 (2.3) years and 48.3% were girls. The prevalence of parasitic infection was 49% with 80% of the infected children exhibiting infestation by *Ascaris lumbricoides* (Table 2). The three groups were homogenous at baseline for age, sex distribution, weight and height and haemoglobin levels (Table 2) however the placebo group portrayed a significantly higher prevalence in *Ascaris lumbricoides* infection.

**Table 2:** Baseline characteristics of children who completed the study by intervention group

Characteristic		Treatment		
	Overall (*n* = 243)	Papaya (*n* = 90)	Albendazole (*n* = 70)	Control (*n* = 83)
Mean ± SD age (years)	8.7 (2.3)	9.1 (2.3)	8.7 (2.2)	9.0 (1.9)
Male, n (%)	167 (51.7)	45 (50)	39 (55.7)	38 (45.8)
Female, n (%)	156 (48.3)	45 (50)	31 (44.3)	45 (54.2)
Mean ± SD Weight (Kg)	24.9 (6.2)	26.4 (7.3)	24.0 (5.4)	24.3 (5.1)
Mean ± SD Height (cm)	126.4 (15.6)	127.8 (20.3)	126.4 (11.4)	127.7 (9.0)
Intestinal Parasites Prevalence (%)				
*Ascaris lumbricoides*		29.9	25.8	43.0
*Trichuris Trichura*		11.3	3.2	3.0
Hookworm		0	1.1	0

### Effects of treatment on parasite infestation and ringworm infections

Table 3 shows the distribution of intensities of infections, expressed as eggs per gram of faeces, before and after treatment. Results for only *Ascaris lumbricoides* will be discussed since infestation by the other parasites was too low to statistically analyse. Geometric mean intensity was significantly reduced (*p* < 0.05) from 209.7 to 75.7 epg (95% confidence interval) during the two-month intervention period in the group that consumed papaya seed fortified porridge. Treatment with albendazole also resulted in a significant reduction in the geometric mean egg count but no significant change (*p* > 0.05) in the control group was observed. The egg reduction rates for the papaya group and the albendazole group was 63.9 and 78.8% respectively.

**Table 3:** *Ascaris lumbricoides* egg counts and ringworm prevalence at baseline and 8 weeks after treatment per treatment group

Outcome	Treatment	Baseline	Follow up	*p*-Value
*Ascaris lumbricoides* Geometric mean (95% CI)	Papaya	209.7 (98.7 to 445.7)	75.7 (36.5 to 157.0)	0.002
	Albendazole	129.5 (37.7 to 444.1)	27.5 (2.2 to 338.1)	0.006
	Control	42.0 (20.0 to 87.9)	56.3 (27.0 to 117.5)	0.657
Ringworm prevalence	Papaya	49 (54.4)	24 (30.0)	0.001
	Albendazole	27 (39.7)	44 (64.7)	0.003
	Control	53 (65.4)	24 (31.2)	< 0.001

Both the papaya group and the control group exhibited a significantly lower (p < 0.05) prevalence of ringworm infection after the 8 weeks treatment period compared to baseline (Table 3). The albendazole group had a significant increase in prevalence of ringworm during follow up compared to baseline.

### Effects on Haemoglobin levels and Anaemia status

Significant improvement in haemoglobin levels was observed at follow up (Table 4) in both the papaya and the albendazole group. No significant change in haemoglobin levels and anaemia status was observed in the control group. A significant high number of previously anaemic children improved to non-anaemic status in the papaya arm as compared to the albendazole and control arm.

**Table 4:** Anthropometric outcomes and Haemoglobin levels of the children before and after 8 weeks intervention

	Treatment	Baseline	Follow up	*P*-Value
MUAC (cm)	Papaya	14.1 (2.8)	14.0 (2.0)	0.511
	Albendazole	12.9 (1.3)	12.7 (1.6)	0.101
	Control	13.0 (1.5)	13.2 (1.7)	0.115
TSF (mm)	Papaya	6.8 (3.4)	8.4 (4.4)	< 0.001
	Albendazole	7.0 (2.5	5.8 (1.9)	< 0.001
	Control	5.3 (1.9)	6.9 (2.5)	< 0.001
Hemoglobin (g/dL)	Papaya	11.5 (1.5)	13.5 (1.2)	< 0.001
	Albendazole	12.8 (1.3)	13.9 (1.0)	< 0.001
	Control	13.2 (1.4)	13.1 (1.2)	0.893

### Nutritional status

There was no significant improvement in BMI for age and MUAC in all the treatment groups. TSF significantly improved in both the papaya and control groups after 8 weeks intervention (Table 4).

## Discussion

The present study suggests that consumption of maize meal porridge fortified with ground seeds of *Carica papaya* and provided as a snack for school children for two months resulted in reduction in intensity of *Ascaris lumbricoides* infection. The overall egg reduction rate of fortified porridge treatment and albendazole was 63.9 and 78.8% respectively. Moreover, consumption of the papaya seed fortified porridge resulted in reduced cases of anaemia and lower infection rates for ringworms.

The reduction in *Ascaris lumbricoides* intensity is consistent with previous studies where dried seeds of *Carica papaya* were used to eliminate intestinal parasites. Okeniyi et al [14], in a randomized clinical study showed that children who received 1 g homey-emulsified dose of papaya seeds showed a parasite clearance rate of 77% after seven days as compared to 17% clearance rate on those who were given honey alone. Our study sought to evaluate the effectiveness of these seeds using a different route of delivery. Porridge made from maize flour is one of the most prevalent traditional school meal snacks in developing countries. Because of its low cost and popularity in schools, the World Food Program has adopted it as a component in school meals and it is often prepared and given as a snack at break time [16]. The idea of the current study was to integrate deworming and micronutrient supplementation, into the school feeding program through the porridge snack so as to offer an affordable, sustainable and home-grown strategy to reduce infestation by intestinal worms while at the same time addressing the issue of malnutrition which is rampant in these resource poor settings [17]. A cross-sectional survey conducted in an urban slum in Kenya demonstrated a relationship between STH infections with iron and Vitamin A deficiency in pre-school children [18]. Micronutrients have also been linked to reduced parasite infestation and re-infection in endemic areas [19, 20].

In this study all the groups were given micronutrient fortified porridge meal. The increase in haemoglobin levels in both the papaya and albendazole groups can be attributed mostly to the micronutrient fortification. This is because the only parasite found to be prevalent in the region was *Ascaris lumbricoides* which is usually not responsible for causing iron deficiency anaemia. In addition, the average haemoglobin levels in the albendazole group and control group were pretty high and also within the normal range, and significant increase was not expected. Previous studies have indicated that deworming alone results in very small non-significant changes in haemoglobin status [21, 22]. Integration of deworming with micronutrient supplementation hence may provide a significant boost to improved child health status.

Analysis of ringworm infection demonstrated a reduction in prevalence in the group that consumed the meal containing papaya seeds. Interestingly, the control group also demonstrated a significant reduction in ringworm infection as compared to the albendazole group. Ringworm is a common contagious infection caused by dermatophytes (fungi). The condition is self-limiting and resultant infections will rarely pose any serious threats to the individual. The illness may however cause a lot of discomfort and disfigurement especially when the lesions are widespread. Though papaya seeds have been reported to have antifungal properties [23, 24] and its topical use for the same is common [25], our observed reduction of ringworms in the control group may suggest that other confounding factors may have also been at play.

Our preliminary results imply that a school porridge meal composed with papaya seeds may be considered as an alternative MDA strategy or a supplement for the large-scale deworming of school children. The introduction of school meal as part of deworming strategy substantially increased school enrolment and retention. The WHO recommended mass drug administration of school children focuses mainly on reducing burden of STH infection in endemic areas. Studies have shown mixed results on the benefits of routine mass deworming alone. For example the Cochrane review [22] showed that routine deworming alone has little or no effect on improving school attendance, hemoglobin status and cognitive ability.

Several drawbacks were evident in our study. The efficacy of papaya seed porridge flour was investigated in an area endemic for roundworms only. Due to their low prevalence in the study population, no evidence was obtained on the efficacy of the papaya fortified porridge on hookworms and whipworms-two of the most prevalent and devastating intestinal parasites infecting children in developing countries. Similarly, our study design was a one site cluster study. Though the participating schools were randomly selected from a cluster of ten schools from the same locality, there might be a need to control for possible other individual and site variables to confirm the outcome or inflate the sample size. Our comparison also was done between schools. There will be a need to confirm through intra-school evaluation to control for possible school differences. Additionally, we could not verify the most effective dosing regimen and frequency. We only did verify the effect of 10 g papaya seed fortified school porridge per day per child for a two month period. The study conducted by Okeniyi et al., [14] among Nigerian children noted an effect within seven days with daily use of only 4 g papaya seeds. Other studies have indicated parasite clearance of 0.6 g and 1.2 g per kilogram body weight over a period of 21 days [26]. There is hence a need to determine the minimal effective dose to inform on future cost reduction strategies in the event that the fortified meal approach is confirmed as a feasible school based deworming strategy.

## Conclusion

This study proves that enhancing a common school porridge with the seeds of *Carica papaya* significantly reduces the burden of infection by *Ascaris lumbricoides*. In comparison, the conventional treatment, albendazole, still had a significantly higher cure rate but less effective on the nutritional status of the children. Papaya seeds are used traditionally as a natural deworming agent. Its antiparasitic potency has also been confirmed scientifically. This study provides a novel approach that uses this knowledge to integrate mass deworming into the school feeding programs in rural Kenya. Papaya seeds are bi-products of the fruit processing industry and their use in this innovation provides a cost effective and environmental friendly strategy for school feeding and deworming programs. If confirmed through further studies, papaya seed fortified school meal may act as an alternative or a supplement to the current mass deworming programs targeting school children in Africa.

## Abbreviations

BMI: Body mass index
CI: Confidence Intervals
DSM: Koninklijke DSM N.V.
Epg: Eggs per gram
FEC: Faecal egg count
g/dL: Grams per decilitre
KEMRI SSC: Kenya Medical Research Institute Scientific Steering Committee
MDA: Mass drug administration
Mg: Milligrams
MUAC: Middle upper arm circumference
NCT: National Clinical Trial
pvalue: Calculated probability
SD: Standard deviation
SPSS: Statistical Package for Social Sciences
STH: Soil transmitted helminths
TEPAD: Terik Essentials Agency Programs for advanced Development
TSF: Triceps skin fold
WHO: World Health organisation
